# S03-3: Building the case: Understanding facilitators and barriers to rolling out Fame in Ireland

**DOI:** 10.1093/eurpub/ckae114.209

**Published:** 2024-09-26

**Authors:** Ruth McCullagh, Dawn Skelton, Frances Horgan

**Affiliations:** Discipline of Physiotherapy, School of Clinical Therapies, University College Cork, Ireland; Research Centre for Health (Reach), School of Health and Life Sciences, Glasgow Caledonian University, Glasgow, United Kingdom; School of Physiotherapy Royal College of Surgeons in Ireland, Dublin, Ireland

## Abstract

Purpose: The Irish Longitudinal Study on Ageing (TILDA) reports that 19% of people aged >50 fall annually. In 2021, the population of > 65s reached 742,300, and is set to reach 1 million within 10 years, with the associated cost of falls se to exceed €2 billion. In 2022, a population health-improvement project, AFFINITY supported the training of Physiotherapists (HSE staff) and Exercise Professionals (community-based private and sole providers) to deliver FaME throughout Ireland, with 120 instructors now trained. However, as previous UK implementation studies have shown, FaME roll-out is complex, with local contexts needing consideration.

Therefore, the purpose of this study is to systematically evaluate and improve the early-adopter sites of FaME in Ireland, thereby providing valuable information for its future implementation in the Irish landscape.

This evaluation study follows the HSE Change Guide; an experience-based co-design action research approach. Three early-adopter sites with varying methods of funding, managing, and delivering FaME have been identified. Using mixed-methods, we are completing the key activities, DEFINE, DESIGN, and DELIVER. We are DEFINing (1) current referral processes to improve equity and reach to people who may fall; (2) the cost, effectiveness (on falls rate, balance, functional independence and well-being), and perceived appropriateness of FaME, and (3) suitable local exercise/activity programmes for participants to maintain independence and function after FaME. From this data, issues in the FaME roll-out will be identified. At co-DESIGN, key stakeholders including PPI representatives, service-users and service-providers, and FaME commissioners, will identify practical local solutions. Finally, in DELIVER, with the local solutions implemented, we will re-explore and re-measure to detect the effects of the change.

We will disseminate the outcomes to public, commissioners and academic parties. The existing UK implementation toolkit will be adapted for the Irish context.

This project will provide valuable information on equitable access, follow-on options, and how best to foster collaborations to ensure the best outcomes for older people and health services. The findings will help to make informed decisions about effective delivery, helping older people to get up and stay active, reducing social isolation, loneliness and dependency.

